# Primary Central Nervous System Tumors in Adolescents: A Population-Based Study on Epidemiology and Clinical Pathways in a Challenging Age Group

**DOI:** 10.3390/curroncol32040222

**Published:** 2025-04-10

**Authors:** Lucia De Martino, Patrizia Piga, Marcella Sessa, Camilla Calì, Camilla Russo, Stefania Picariello, Nicola Onorini, Pietro Spennato, Lucia Quaglietta, Maria Vittoria Donofrio, Giuseppe Cinalli, Francesco Vetrano, Fabio Savoia

**Affiliations:** 1 Neurooncology Unit, Department of Pediatric Oncology, Santobono-Pausilipon Children’s Hospital, 80129 Naples, Italy; l.demartino1@santobonopausilipon.it (L.D.M.); s.picariello@santobonopausilipon.it (S.P.); l.quaglietta@santobonopausilipon.it (L.Q.); 2 Childhood Cancer Registry of Campania, Santobono-Pausilipon Children’s Hospital, 80129 Naples, Italy; p.piga@santobonopausilipon.it (P.P.); m.sessa@santobonopausilipon.it (M.S.); c.cali@santobonopausilipon.it (C.C.); f.vetrano@santobonopausilipon.it (F.V.); f.savoia@santobonopausilipon.it (F.S.); 3 Pediatric Neuroradiology Unit, Department of Pediatric Neurosciences, Santobono-Pausilipon Children’s Hospital, 80129 Naples, Italy; c.russo3@santobonopausilipon.it; 4 Pediatric Neurosurgery Unit, Department of Pediatric Neurosciences, Santobono-Pausilipon Children’s Hospital, 80129 Naples, Italy; n.onorini@santobonopausilipon.it (N.O.); g.cinalli@santobonopausilipon.it (G.C.); 5 Pathology Unit, Santobono-Pausilipon Children’s Hospital, 80129 Naples, Italy; v.donofrio@santobonopausilipon.it

**Keywords:** adolescents, CNS tumors, clinical pathways, cancer registry, epidemiology

## Abstract

Background: Oncological care of adolescent patients is often inconsistent, as they frequently fall between pediatric and adult services. The Childhood Cancer Registry of Campania (CCRC) is the Italian largest population-based registry specializing in children 0–19 years old, with a target population of approximately 1.1 million inhabitants. Material and Methods: This report presents epidemiological indicators and clinical pathways on primary brain tumors in adolescents (15–19 years) from the Campania region. Results: Over the study period (2008–2020), the cohort included 219 adolescents with newly diagnosed central nervous system (CNS) tumors with an annual average incidence rate (IR) of 48.9 cases per million/year. The 5-year observed survival rate after diagnosis of CNS tumor was 84.8%. Overall, the most common tumor site was the pituitary gland and craniopharyngeal duct, representing 22.4% of all tumors. The most frequently occurring malignant primary CNS tumor was germinoma, while the most common non-malignant tumor was pituitary adenoma. Most patients were referred to adult services and nearly half migrated outside the region to receive cancer care. Conclusions: Challenges in the care of adolescent oncology patients include limited access to specialized care, difficulties in transitioning from pediatric to adult institutions, distinct tumor biology, and the underrepresentation of adolescents in clinical trials. The care of adolescents with CNS tumors is fragmented across institutions and significant variations in practice exist between adult and pediatric practitioners.

## 1. Introduction

Primary CNS tumors arising in adolescents differ significantly from those in children and adults, not only in their morphological and molecular characteristics but also in their clinical management, including diagnostic procedure, neurosurgical approach, and medical treatment. From an epidemiological perspective, indicators for this age group are often either underrepresented or combined with those of pediatric or adult populations (sometimes classified as adolescents and young adults, AYA), leading to a significant loss of valuable information [1]. While survival rates for cancer diagnoses during adolescence have improved over the past decades in both international and national data [2,3], this trend has not been as evident for CNS tumors [1,4]. One of the challenges in the management of adolescents with primary CNS tumors is that the histopathologies of such tumors in adolescents differ from those seen in either children or older adults. In addition, oncological care of adolescent patients is often erratic as they frequently fall between pediatric and adult services, which hinder their recruitment and participation in clinical trials [5,6]. The Childhood Cancer Registry of Campania (CCRC) is the largest Italian population-based registry focused on children 0–19 years of age, with a target population of approximately 1.1 million inhabitants. The aim of our study is to analyze the epidemiology of primary CNS tumors diagnosed in patients between 15 and 19 years of age in the Campania region, as well as to explore their pathway of care.

## 2. Materials and Methods

### 2.1. Data Collection

The study includes cases of primary CNS tumors diagnosed in adolescents (age range 15–19 years) residing in Campania, between 2008–2020. The data was provided by the Childhood Cancer Registry of Campania, a population-based registry, specializing in oncological surveillance of tumors in individuals aged 0–19 years. The registry is located at Santobono-Pausilipon Children’s Hospital, the regional reference center for the diagnosis and treatment of pediatric tumors.

For case registration, the main data sources commonly used by cancer registries were employed, including hospital discharge records, pathology reports, and death certificates. Additionally, supplementary sources such as medical records, molecular biology and outpatient reports were utilized. These supplementary data sources are particularly important for intracranial and intraspinal tumors, as a significant proportion of these cases is not pathologically confirmed. In accordance with international cancer registry registration rules, our study includes both histologically confirmed and unconfirmed CNS tumors (ENCR basis of diagnosis recommendations, https://www.encr.eu/sites/default/files/Recommendations/ENCR%20Recommendation%20BoD_Oct2022_EN_241022.pdf accessed on 15 December 2024).

The recorded data include demographic variables; tumor morphology and topography according to the International Classification of Diseases for Oncology, third edition (ICD-O-3) (5); date of diagnosis; tumor behavior (benign, borderline, malignant); basis of diagnosis; and date of last contact. The registered cases were transcoded into the International Classification of Childhood Cancer, third edition (ICCC) [7,8]. The study is not limited to cases classified under the third ICCC-3 category “CNS and miscellaneous intracranial and intraspinal neoplasms” but extends to all intracranial and intraspinal tumors, including hematolymphoid tumors involving the CNS and germ cell tumors, based on topographic criteria (ICD-O-3 codes C70–C72, C75.1, C75.2). There are over 100 histologically distinct types of primary CNS tumors, which are periodically reviewed by neuropathologists and published by the World Health Organization (WHO) in Classification Reports known as “Blue Books”. Blue Books are published for all cancer sites by WHO and utilize the ICD-O-3) to assign histology, behavior, and site codes. This report uses the 2007 and 2016 WHO Classification of Tumors of the Central Nervous System as the guiding framework for reporting.

It is important to note that the report on lymphomas and hematopoietic neoplasms contained here refer only to those neoplasms arising within the brain and CNS, based on the ICD-O-3 topography codes. Following the 2007 and 2016 World Health Organization (WHO) guidelines, WHO grades were assigned to all tumors (grade I through grade IV) based on predicted clinical behavior [9,10]. However, it is not possible to determine the WHO grade when the tumor diagnosis is made exclusively on neuroimaging without histopathological confirmation Additionally, certain tumor types, including pituitary tumors, are often not assigned a WHO grade.

In this study we describe: (1) the pathway of care of adolescents with primary CNS tumors, identifying the site of diagnosis (pediatric neurosurgery, adult neurosurgery, other centers) and treatment (pediatric oncology, adult oncology); (2) migration outside Campania region; and (3) fragmentation in the delivery of healthcare across neurosurgical centers.

### 2.2. Statistical Analysis

The incident cases were described using absolute and relative frequencies, according to the ICCC-3 classes. For each diagnostic subclass, the male–female ratio, percentage of microscopic diagnoses, and incidence rate were calculated. The overall age-specific incidence rates per million/year were calculated using the person-years provided by the National Institute of Statistics (ISTAT). Survival analysis was conducted using a cohort approach, which was used to calculate the 5-year observed survival (OS) for patients diagnosed during 2008–2017, as there was at least a 5-year follow-up available for all patients. We calculated OS with 95% confidence intervals, stratified by gender, behavior, ICCC class, and diagnosis period (five-year intervals). The survival function was estimated using the Kaplan–Meier method, and differences between groups were assessed using the log-rank test. Two-sided tests with p less than 05 were considered statistically significant. Analysis was performed using Stata 17 (Stata Corporate).

The fragmentation of providers (neurosurgical centers) for each class of diagnosis is modeled on a standard Herfindahl–Hirschman concentration index (HHI%). The formula is as follows:

$$HHI\%=\left(\sum_{i=1}^{N}MS_{i}^{2}\right)\times 100$$

where MS is the portion of patients sharing providers, and N is the number of providers. This HHI% ranges from 100%, corresponding to having all care delivered by a single provider (monopoly), and fragmentation approaching 0% if the patient’s care was split equally among a very large number of providers (perfect competition).

## 3. Results

Between 2008 and 2020, the study cohort included 219 adolescents (111 males, 50,7%), aged 15 to 19 years, with newly diagnosed CNS tumors. Of these, 74.9% had tumors that were confirmed histopathologically. A larger proportion of malignant tumors were confirmed histopathologically (91.9%), compared with non-malignant tumors (65.5%). For the 55 unconfirmed CNS tumor diagnoses (25.1%), the diagnosis was based on neuroimaging (*n* = 45, 20.5%) and specific tumor markers (*n* = 10, 4.6%), as per ENCR recommendations (Table 1).

### 3.1. Incidence: Newly Diagnosed Primary CNS Tumors in Adolescents, 2008–2020

Between 2008 and 2020, primary CNS tumors (both malignant and non-malignant) in our cohort of adolescents have an annual average incidence rate (IR) of 48.9 cases per million/year (Table 2).

In diagnostic group III, i.e., “CNS and miscellaneous intracranial and intraspinal neoplasms” (encompassing both malignant and non-malignant), the IR was 42.0. This class is the third-most common cancer in individuals aged 15–19 years, after carcinomas and lymphomas. The incidence of malignant group III CNS tumors (ICD-O-3 behavior code of /3) was 12.3 per million/year compared to 29.7 per million/year for non-malignant tumors (ICD-O-3 behavior code of /0 or /1).

The rate of all primary malignant and non-malignant CNS tumors was higher in females than in males (46.4 vs. 37.8 per million/year, respectively). Of note, no significant sex difference was observed in the incidence of malignant CNS tumors within diagnostic group III (12.6 in males vs. 11.9 in females). Germinomas had an IR of 2.7 per million/year, with 12 new cases registered over the 13-year study period.

### 3.2. Survival: From Diagnosis to 5 Years in Adolescents, 2008–2017

The 5-year observed survival rate after diagnosis of a primary malignant or non-malignant CNS tumor was 84.8%. Survival probability for CNS tumors varies widely depending on the tumor type, ranging from 50% for gliomas and 75% for embryonal tumors (including medulloblastoma), to 85.7% for ependymomas, 90% for germ cell tumors and 93.9% for other specific CNS tumors, such as pituitary adenomas, which have an excellent prognosis.

**Figure 1:**
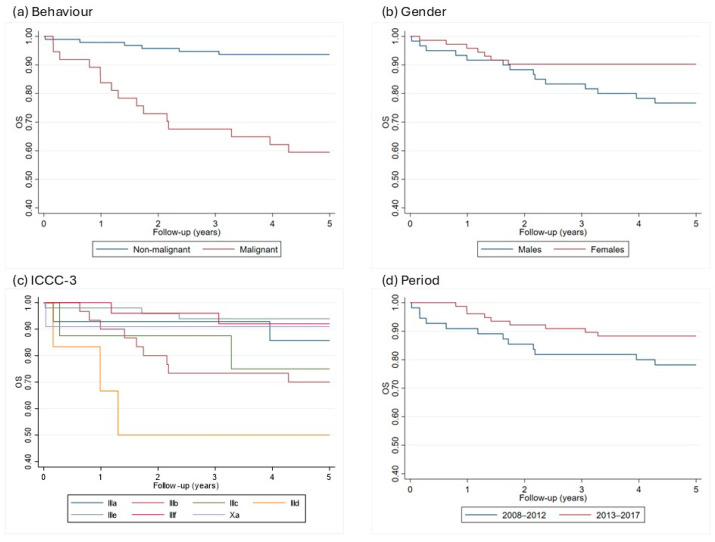
5-years Kaplan–Meier curves by behavior (**a**), gender (**b**), ICCC subgroups (**c**), and incidence period (**d**) in the Campania region, period 2008–2017. ICCC-3 = International Classification of Childhood Cancer, OS = overall survival.

In diagnostic group III, i.e., “CNS and miscellaneous intracranial and intraspinal neoplasms”, survival rates also differ by tumor behavior; in fact malignant tumors have a survival rate of 59.5%, significantly lower than the 93.7% of non-malignant tumors. When considering all tumor types, females show a better prognosis, with a 5-year survival rate of 90.3%, compared to 76.7% in males. This difference is partially attributed to the different case-mix between the genders, where females have a higher incidence of tumors with a better prognosis, such as pituitary adenomas (Figure 1, Table 3).

### 3.3. Distributions of Tumors by Behavior, Site and Histology

**Figure 2:**
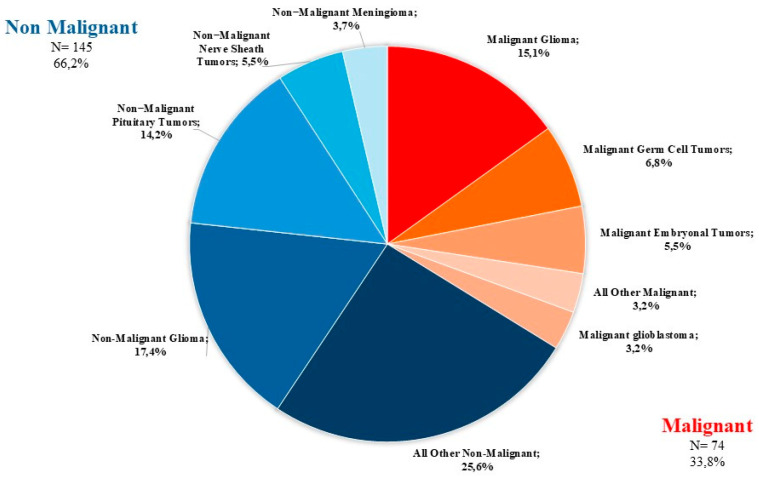
Distribution of primary brain and other CNS tumors by behavior (thirteen-year total= 219; annual average cases = 17) in the Campania region, period 2008–2020. CNS= central nervous system.

The distribution of all 219 primary brain and other CNS tumors reported between 2008–2020 categorized by behavior (74 malignant; 145 non-malignant) is presented in Figure 2.

**Figure 3:**
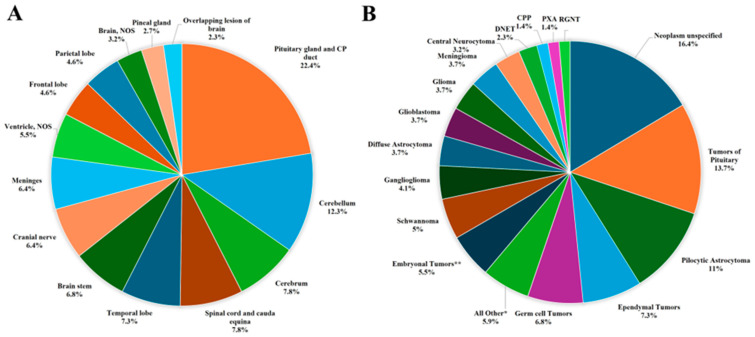
Distribution of primary brain and other CNS tumors (malignant and non-malignant combined), by (**A**) site and (**B**) histology (thirteen-year total = 219; annual average cases = 17) in the Campania region, period 2008–2020. CNS = central nervous system; NOS = not otherwise specified; CP = Craniopharyngeal; DNET = Dysembryoplastic Neuroepithelial Tumor; CPP = Choroid Plexus Carcinomas; PXA = Pleomorphic Xanthoastrocytoma; RGNT = Rosette-forming Glioneuronal Tumor; * Includes all histologies with frequency < 1%, including: craniopharyngioma (2), lymphoma (2), astroblastoma (1), anaplastic (1) and fibrillary (1) astrocytoma, Langerhans cell histiocytosis (1), malignant peripheral nerve sheath tumor (1), meningeal melanomatosis (1), oligodendroglioma (1), subepnedymal gian cell astrocitoma (1), diffuse intrinsic pontine glioma (1); ** Includes medulloblastoma (11), atypical teratoid-rabdoid tumor (1).

**Figure 4:**
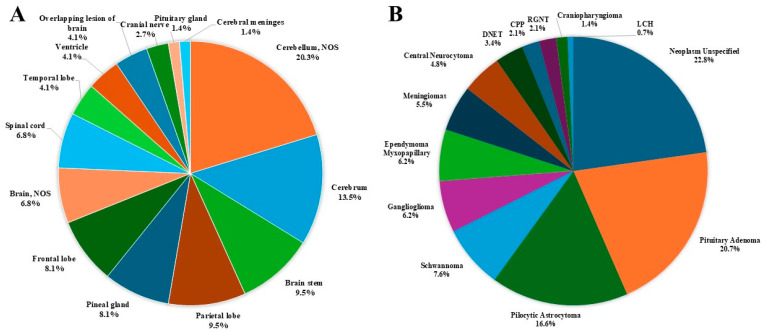
Distribution of malignant primary brain and other CNS tumors, by (**A**) site and (**B**) histology (thirteen-year total = 74; annual average cases = 6) in the Campania region, period 2008–2020. CNS = central nervous system, NOS = not otherwise specified; CPP = Choroid Plexus Papilloma; DNET = Dysembryoplastic Neuroepithelial Tumor; LCH = Langherans Cell Histiocitosis; RGNT = Rosette-forming Glioneuronal Tumor.

**Figure 5:**
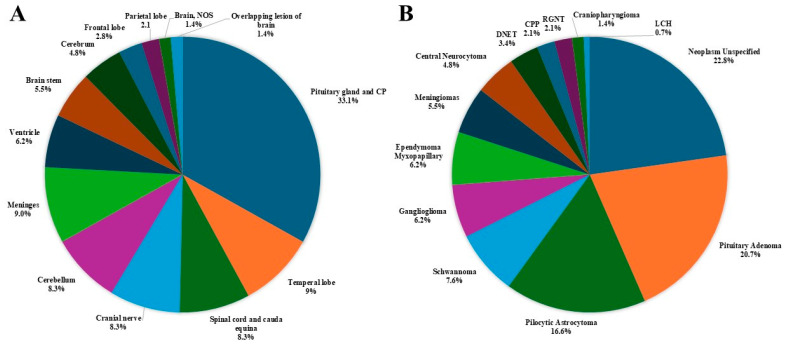
Distribution of non-malignant primary brain and other CNS tumors, by (**A**) site and (**B**) histology (thirteen-year total = 145; annual average cases = 11) in the Campania region, period 2008–2020. CNS = central nervous system; NOS = not otherwise specified; CPP = Choroid Plexus Papilloma; DNET = Dysembryoplastic Neuroepithelial Tumor; LCH = Langherans Cell Histiocitosis; RGNT = Rosette-forming Glioneuronal Tumor.

The distribution of all primary brain and other CNS tumors by site and histology is presented in Table 1 and Figure 3. Distributions for malignant and non-malignant tumors by site and histology are presented in Figure 4 and Figure 5, respectively.

- The most common tumor site was the pituitary gland and craniopharyngeal duct, representing 22.4% of all tumors.
- Frontal (4.6%), temporal (7.3%), and parietal (4.6%) lobes combined represented 16.5% of all tumors.
- The cerebellum accounted for 12.3% of all tumors.
- The most frequently reported histology overall was non-malignant pituitary tumors (13.7%), followed by pilocytic astrocytoma (11%) and ependymal tumors (7.3%).
- For malignant tumors, parietal (9.5%), frontal (8.1%), temporal (4.1%) lobes accounted for 21.7% of cases (Figure 4).
- The most common malignant CNS tumors were germ cell tumors (20.3%), followed by embryonal tumors (16.2%). Among embryonal tumors, medulloblastoma and atypical teratoid rhabdoid tumor (ATRT) accounted for 91.7% and 8.3%, respectively.
- For non-malignant tumors, 33.1% were located in the pituitary gland and craniopharyngeal duct (CP).
- The most common histology among non-malignant tumors was pituitary adenoma (20.7%), followed by pilocytic astrocytoma (16.6%).

### 3.4. Pathway of Care

The pathway of care of adolescents is described in Table 4.

**Figure 6:**
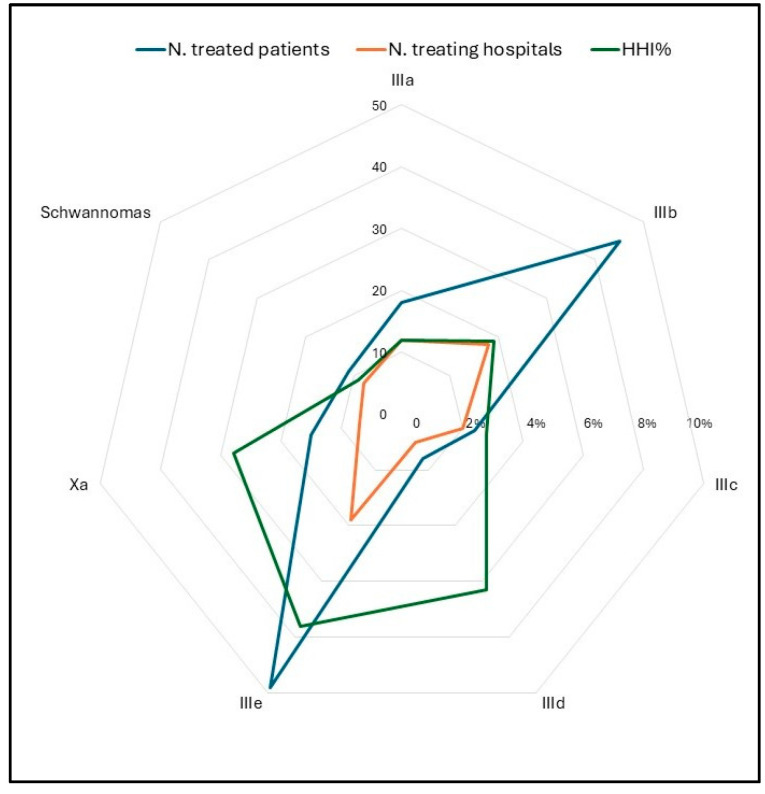
Radar chart of the number of treated patients (blue), number of treating hospitals (orange), and HHI% (green) by ICCC subgroups, period 2008–2020. HHI = Herfindahl–Hirschman concentration index; ICCC = International Classification of Childhood Cancer.

One hundred seventy-five adolescents (79.9%) were referred to a neurosurgical unit for diagnosis and/or neurosurgical treatment. The remaining patients (20.1%) were referred to other medical units (internal medicine, endocrinology, neurology). In 65.3% of cases, neurosurgery was managed by adult units. Fifty-seven adolescent patients (26%) were referred to specialized pediatric oncology units after their initial presentation. One hundred five (57.1%) patients underwent diagnosis and/or neurosurgical procedures in the Campania region. The remaining ninety-four (42.9%) migrated outside of the region. The distribution of hospitals where neurosurgical procedures were performed revealed a high degree of fragmentation. The HHI was 5.6% for malignant tumors and 4.2% for non-malignant tumors. The highest HHI% is observed for class IIIe, i.e., “Other specified CNS tumors”, primarily due to the fact that pituitary adenomas are predominantly treated in two specialized centers (Figure 6, Table 3).

## 4. Discussion

Campania is the second-largest region in Italy in terms of adolescent population, accounting for 11% of the national population aged 15–19 years. This study provides a comprehensive epidemiological overview of CNS tumors, including a detailed morphological and topographical description. The incidence rates observed in Campania, especially for malignant CNS tumors, are comparable and slightly lower than those reported in previous European studies [11]. A higher incidence of intracranial germinomas is observed. The literature describes higher incidence rates in some Asian countries compared to North America and Europe [12]. However, incidence estimates for these diagnoses in adolescents are very rare, limiting the possibility of comparisons. Similarly, survival rates are also largely consistent with those reported in studies from the pool of European cancer registries, with an estimated probability of about 59.4% for malignant tumors [13]. Overall, our results do not show a statistically significant increase in five-year survival during the study period. Similarly, other studies with greater statistical power have not observed an improvement in this age group [2]. Clinical pathways have also been analyzed for tumors characterized by high mortality and morbidity. These pathways are particularly complex for CNS tumors, as the diagnostic and therapeutic processes require coordinated efforts among neurosurgeons, oncologists, neuroradiologists, and other specialists. This complexity is heightened when treating adolescents during transition from pediatric to adult care. Most patients were referred to adult services and nearly half of patients migrate to other regions to receive cancer care. Managing the clinical care of adolescents with CNS tumors and ensuring access to optimal treatment remains a significant challenge. Several reports on adolescents with cancers have shown worse survival rates compared to children with different neoplasms, including leukemias and lymphomas, astrocytomas, and bone and soft tissue sarcomas [14,15,16]. Disparities in access to cancer services and differences in treatments and quality of care delivered are believed to contribute to this result. Our experience highlights how adolescents are often arbitrary referred to either pediatric or adult medical oncologists based solely on age due to strict upper age limits set by pediatric hospitals, inadequate collaboration between pediatric and adult medical oncologists, and a shortage of organized healthcare networks focusing on adolescents. Policies aiming to improve healthcare productivity often focus on reducing care fragmentation, which occurs when services are spread across many providers, potentially making coordination difficult [16,17]. Hence, as observed in other European population-based studies, promoting the centralization of diagnostic and therapeutic processes is crucial. Strengthening expertise in pathology and improving access to molecular diagnostics are essential for enhancing diagnostic accuracy.

Beyond molecular testing, interpretation of relevant variants to identify and access targeted therapies requires input from both adult and pediatric specialists across neuro-oncology, radiation-oncology, neurosurgery, neuroradiology, and molecular pathology. Centralization and multidisciplinary discussions also ensure that all adolescents have access to the most appropriate and advanced treatments, with a positive impact on prognosis [16,18]. In our cohort of adolescents, however, we did not identify a diagnostic and therapeutic pathway for CNS tumors. The values obtained from the HHI index indicate the high fragmentation of care for our adolescents with CNS tumors across various institutions. In Italy, the AIEOP Committee on Adolescents launched various initiatives to improve the quality of care for this age group. Ferrari et al. reported that the percentage of adolescents treated at AIEOP centers has increased over the years, rising from 10% in 1989–2006 to 28% in 2007–2012, and reaching 37% in 2013–2017 [18]. In our study, only twenty-six (26%) patients were treated at a pediatric oncology center during the observation period (2008–2020). CNS tumors are one of the most critical cancers since they affect nearly every aspect of the patients’ lives. Many adolescents diagnosed with brain or spinal cord tumors, were previously healthy people, with active and productive lives. Sadly the disease often leads to significant mortality and morbidity due to the irreversible damage to the CNS. Since CNS tissue has limited regenerative abilities, most tumor related brain injury, whether direct or indirect, is permanent and significantly affects patients’ well-being with a profound impact on their personal, social and financial lives. In recent years, there has been growing recognition within the research, clinical care, and patient advocacy communities of the significant lack of attention and resources directed toward adolescent cancer patients and survivors. A position paper from the AYA Working Group of the European Society for Medical Oncology (ESMO) and the European Society for Pediatric Oncology (SIOPE) highlighted the current situation and challenges in AYA cancer care in Europe, and the urgent need for solutions and interventions [5]. Survival rates of AYA populations are higher when these patients are treated on therapeutic trials and at specialized centers [19]. The 2021 WHO Classification for CNS Tumors (WHO CNS5) emphasizes the importance of integrated molecular and histologic characterization in adolescent tumors, particularly for glioma, medulloblastoma, and ependymoma [20]. Since 2023, a pediatric and adolescent diagnostic-therapeutic care pathway is available in Campania, which recommends that adolescent up to 18 years of age should be referred to pediatric neuro-oncology specialists and discussed within a multi-disciplinary tumor board. Future direction may aim to develop an adolescent cancer center dedicated to the diagnosis, treatment and follow-up of this population to improve oncological outcomes and to manage many age-related issues including onco-fertility, cancer survivorship, neurocognitive and physical rehabilitation, and psychosocial support.

There are limitations to the present study that should be acknowledged owing to its retrospective design. First, beyond survival, the heterogeneity of tumors and the small numbers did not allow us to perform a detailed analysis of prognostic factors in this selected cohort of adolescents. Second, cancer treatment protocols are not reported in the present study. Additionally, since the studies are based on cancer registry data, the information is not fully up to date, with the last year of recruitment being 2020. However, despite these limitations, this population-based study offers validated data from a specialized cancer registry, adhering to established standards of accuracy and case completeness. These data raise awareness on the need for implementation of current knowledge and clinical practice to improve outcomes for adolescents with CNS tumors.

## 5. Conclusions

The spectrum of neuro-oncological diagnoses in adolescents encompasses both pediatric and adult entities. The care of our adolescents with CNS tumors is fragmented across institutions. Adolescents with CNS tumors should be recognized as a distinct group within neuro-oncology to establish standards of practice, to address targeted clinical trials, and provide personalized care to improve patient outcomes.

## Figures and Tables

**Table 1:** Base of diagnosis of histologically unconfirmed CNS tumors. Campania region, period 2008–2020.

*n* = 55 (25.1%)	Neuroimaging (*n*, %)	Specific Tumor Markers (*n*, %)
Non-malignant neoplasm (80,000, 80,001)	33 (100%)	0
Malignant neoplasm (80,003)	3 (100%)	0
Pituitary adenoma (82,710, 82,720)	5 (33.3%)	10 (66.7%)
Malignant glioma (93,803)	3 (100%)	0
Subependymal giant cell astrocytoma (93,841)	1 (100%)	0

CNS = central nervous system.

**Table 2:** Number of cases, percentage, male/female ratios (M/F), WHO grade, percentage of microscopically confirmed diagnoses and age-specific incidence rates, by diagnostic subgroup (ICCC-3). Campania region, period 2008–2020.

	Intracranial and Intraspinal Tumors International Classification of Childhood Cancer—3rd ed	ICD-O-3a Histology Code	Sex (M/F)	N° of Newly Diagnosed Tumors	%	WHO Grade	Histologically Confirmed (%)	Incidence Rate **
IIIa Ependymoma and choroid plexus tumor			13/6	19	10.1		100	4.2
	Myxopapillary ependymoma *	9394/1	5/3	8	4.3	I	100	1.8
	Choroid plexus papilloma *	9390/0	2/1	3	1.6	I	100	0.7
	Ependymoma, other and NOS	9391/3, 9392/3	6/2	8	4.3	II	100	1.8
IIIb Astrocytomas			27/19	46	24.5		100	10.3
	Pilocytic astrocytoma *	9421/1	14/10	24	12.8	I	100	5.4
	Pilomyxoid astrocytoma	9425/3	1/1	2	1.1	I	100	0.4
	Fibrillary astrocytoma	9420/3	1/0	1	0.5	II	100	0.2
	Anaplastic astrocytoma	9401/3	1/0	1	0.5	III	100	0.2
	Subependymal astrocytoma *	9384/1	0/1	1	0.5		0	0.2
	Pleomorphic xanthoastrocytoma	9424/3	0/1	1	0.5		100	0.2
	Glioblastoma and variants	9440/39,441/3	6/2	8	4.3	IV	100	1.8
	Astrocytomas, NOS	9400/3	4/4	8	4.3		100	1.8
IIIc Intracranial and intraspinal embryonal tumor			8/5	12	6.4		100	2.7
	Medulloblastoma, variants	9470/3, 9474/3	6/2	8	4.3	IV	100	1.8
	Desmoplastic/nodular medulloblastoma		1/2	3	1.6	IV	100	0.7
	Atypical teratoid/rhabdoid tumor	9508/3	1/0	1	0.5	IV	100	0.2
IIId Other gliomas			2/9	11	5.9		73	2.5
	Oligodendroglioma	9450/3	0/1	1	0.5	II	100	0.2
	Astroblastoma	9430/3	0/1	1	0.5		100	0.2
	Glioma NOS (excl.optic nerve)	9380/3, 9385/3	2/7	9	4.8		56	2.0
IIIe Other specified CNS tumors			23/41	64	34.0		77	14.3
	Dysembryoplastic neuroepithelial tumor *	9413/0	2/3	5	2.7	I	100	1.1
	Gangliocytomas, ganglioglioma *	9505/1	4/5	9	4.8	I	100	2.0
	Meningioma, non-malignant *	9531/0, 9532/0, 9533/0, 9537/0, 9538/1, 9539/1	7/1	8	4.3	I	100	1.8
	Craniopharyngioma *	9351/1	2/0	2	1.1	I	100	0.4
	Central neurocytoma *	9506/1	1/6	7	3.7	II	100	1.6
	Papillary glioneuronal tumor *	9509/1	0/3	3	1.6	I	100	0.7
	Pituitary tumors *	8270/0, 8271/0	7/23	30	16.0	-	50	6.7
IIIf Other specified CNS tumors			14/22	36	19.1		0	8.0
	Malignant	8000/3	1/2	3	1.6	-	0	0.7
	Non-malignant *	8000/0, 1	13/20	33	17.6	-	0	7.4
Total malignant CNS tumors			29/26	55	29.3		89	12.3
Total malignant & non-malignant CNS tumors			87/101	188			71	42.0
Other intracranial and intraspinal tumors								
II Lymphomas and others reticuloendothelial cell tumors			3/0	3	9.7		100	0.7
	Malignant lymphoma, large B-cell, diffuse, NOS	9680/3						
	Malignant lymphoma, large B-cell, diffuse, immunoblastic, NOS	9683/3						
	Langerhans cell histiocytosis *	9751/1						
IXb Fibrosarcomas, peripheral nerve sheath tumors			1/0	1	3.2		100	0.2
	Nerve sheath tumors	9540/3	1/0	1	3.2		100	0.2
Xa Intracranial and intraspinal germ cell tumors			15/0	15	48.4		100	3.3
	Dysgerminoma	9060/3	1/0	1	3.2		100	0.2
	Germinoma	9064/3	12/0	12	38.7		100	2.7
	Mixed germ cell tumors	9085/3	2/0	2	6.5		100	0.4
XId Melanomas			1/0	1	3.2		100	0.2
	Meningeal melanomatosis	8720/3	1/0		3.2		100	0.2
No ICCC coded			5/7	11	35.5		100	2.5
	Schwannomas *	9560/0	4/7	11	35.5		100	2.5
Total other intracranial and intraspinal tumors			24/7	31			100	6.9

ICD-O-3a = International Classification of Diseases for Oncology, third edition, M = male, F = female, WHO = World Health Organization, NOS, CNS= central nervous system, ICCC = International Classification of Childhood Cancer; * non malignant tumors; ** per million inhabitants/year.

**Table 3:** 5-years observed survival, 95% confidence interval and *p*-value by gender, behavior, incidence period, and ICCC-3 subgroups in the Campania region, period 2008–2017.

		5-Year Survival			Equality of Survivor Functions
		OS%	95% CI		Log-Rank Test (*p* Value)
Sex					
	Males	76.7	63.8	85.5	
	Females	90.3	80.7	95.2	<0.05
Behavior					
	Malignant	59.5	42.0	73.2	
	Non-malignant	93.7	86.5	97.1	<0.001
Period					
	2008–2012	78.2	64.8	87.0	
	2013–2017	88.3	78.7	93.7	0.11
ICCC-3					
	IIIa Ependymoma and choroid plexus tumor	85.7	53.9	96.2	
	IIIb Astrocytomas	70.0	50.3	83.1	
	IIIc Intracranial and intraspinal embryonal tumor	75.0	31.5	93.1	
	IIId Other gliomas	50.0	11.1	80.4	
	IIIe Other specified CNS tumors	93.9	82.2	98.0	
	IIIf Other unspecified CNS tumors	92.0	71.6	97.9	
	Xa Intracranial and intraspinal germ cell tumors	90.9	50.8	98.7	

ICCC-3 = International Classification of Childhood Cancer, OS = overall survival, CI = confidential interval, CNS = central nervous system.

**Table 4:** Centre of diagnosis of primary CNS tumors in adolescents. Campania 2008–2020.

	N (%)	Histologically Confirmed
Adult neurosurgery Campania region Extra regional	143 (65.3%) 79 (55.2%) 64 (44.8%)	133 (93%) 71 (89.9%) 58 (90.6%)
Pediatric neurosurgery Campania region Extra regional	32 (14.6%) 17 (53.1%) 15 (46.9%)	30 (93.7%) 14 (82.3%) 15 (100%)
Other medical unit Campania region Extra regional	44 (20.1%) 29 (65.9%) 15 (34.1%)	0 (0%)

CNS = central nervous system.

## Data Availability

The original contributions presented in this study are included in the article. Further inquiries can be directed to the corresponding author(s).

## Abbreviations

The following abbreviations are used in this manuscript:

CCRC	Childhood Cancer Registry of Campania
CNS	Central nervous system
AYAs	Adolescents and young adults
AIEOP	Associazione Italiana Ematologia Oncologia Pediatrica
ICD-O	Classification of Diseases for Oncology
ICCC	Classification of Childhood Cancer
WHO	World Health Organization
OS	Observed survival
IR	Incident rate
CPP	Choroid plexus papilloma
ESMO	European Society for Medical Oncology
SIOPE	European Society for Pediatric Oncology
